# Letter to the Editor: “Dry Eye Disease after Cataract Surgery: Study of its Determinants and Risk Factors”

**DOI:** 10.4274/tjo.galenos.2020.83893

**Published:** 2020-12-29

**Authors:** Nir Erdinest, Naomi London

**Affiliations:** 1 Hadassah-Hebrew University Medical Center, Department of Ophthalmology,; 2 Private practice, Jerusalem, Israel

**Keywords:** Dry eye disease, cataract surgery

## Dear Editor,

This letter is regarding the article titled “Dry Eye Disease after Cataract Surgery: Study of its Determinants and Risk Factors”.^1^ We read this article with great interest and thank the authors for providing an excellent demonstration that phacoemulsification and small-incision cataract surgery can cause dry eye. The authors included in this study patients that pre-surgery were completely asymptomatic and without clinical dry eye signs and this study exhibits that the elements of the surgery itself indeed were the cause of dry eye development, which peaked one week post-surgery and subsided around one month post-surgery. It was apparent the authors appreciate the importance of eliminating the patients with dry eye from this study and their workup was thorough. We would like to stress the importance of treating those with clinical signs with or without symptoms prior to surgery, as an unstable tear film can profoundly undermine a successful outcome, for example, by affecting keratometry and topography readings and consequently the calculation of the intraocular lens.^2^ One important element to add to the risk factors that was not mentioned, are the lid retractors that are utilized during surgery. Ptosis is a well-documented possible post-surgery complication caused by a number of factors including anesthesia myotoxicity and use of a lid speculum.^3^ Studies have noted a lower lid laxity for up to three months after phacoemulsification,^4^ particularly relevant over age 70 but important to mention considering the age group of this study as well. These retractors can slightly change the position of the lower lid, which potentially affects proper blink. The lower punctum’s location moves slightly anteriorly, which potentially influences tear film and drainage. These temporary and sometimes permanent alterations can directly impact dry eye development. Considering the high presentation of dry eye postoperatively, we agree with the authors that the addition of topical lubrication would be helpful. Perhaps in cases with clinical signs, even if asymptomatic, an additional consideration would be to preemptively suggest treatment to improve the tear film prior to surgery with recognized therapies such as topical cyclosporine, which has been shown to improve visual acuity and contrast sensitivity in post cataract surgery patients with multifocal intraocular implants.^5^
